# What Motivates Us for Work? Intricate Web of Factors beyond Money and Prestige

**DOI:** 10.1371/journal.pone.0132641

**Published:** 2015-07-15

**Authors:** Nadja Damij, Zoran Levnajić, Vesna Rejec Skrt, Jana Suklan

**Affiliations:** 1 Faculty of Information Studies in Novo mesto, Novo mesto, Slovenia; 2 School of Advanced Social Studies, Nova Gorica, Slovenia; 3 Faculty of Computer and Information Science, University of Ljubljana, Ljubljana, Slovenia; 4 Varsi d.o.o., Ljubljana, Slovenia; 5 Interdisciplinary Doctoral Study Programme in Statistics, University of Ljubljana, Ljubljana, Slovenia; University of Maribor, SLOVENIA

## Abstract

Efficiency at doing a certain task, at the workplace or otherwise, is strongly influenced by how motivated individuals are. Exploring new ways to motivate employees is often at the top of a company’s agenda. Traditionally identified motivators in Western economies primarily include salary and prestige, often complemented by meaning, creation, challenge, ownership, identity, etc. We report the results of a survey conducted in Slovenia, involving an ensemble of highly educated employees from various public and private organizations. Employing new methodologies such as network analysis, we find that Slovenians are stimulated by an intricate web of interdependent factors, largely in contrast to the traditional understanding that mainly emphasizes money and prestige. In fact, these key motivators only weakly correlate with the demographic parameters. Unexpectedly, we found the evidence of a general optimism in Slovenian professional life - a tendency of the employees to look at the “bright side of things”, thus seeing more clearly the benefits of having something than the drawbacks of not having it. We attribute these particularities to Slovenian recent history, which revolves around gradually embracing the Western (economic) values.

## Introduction

Whether in our private or professional life, every day we complete a certain amount of tasks, some of which are more or less pleasurable to do. Of course, when motivated or stimulated to do certain tasks, we often complete them faster, better and without procrastination, even when the tasks themselves are not very pleasurable. Motivation in general comes from a wide range of personal or social factors, such as financial compensation (salary), recognition by the colleagues or superiors (prestige), or satisfaction coming from personal achievements. It comes as no surprise that employers and companies are systematically seeking new ways to stimulate their employees towards being more productive and happier at the same time [1]. In conditions of radical social and cultural changes, in particular those related to the emerging knowledge economy, enterprises are facing new challenges to motivate and retain key workforce, which is the focus factor of competitiveness in the market.

What motivates and what demotivates (inhibits) the individuals in our society has been widely investigated through the framework of motivation theory. Herzberg [2], maintains that “it is only when one has a generator of one's own that we can talk about motivation”. According to Grant [3] the motivation of employees significantly boosts the levels of persistence, productivity and work performance. In fact, there are several distinct theories seeking to shed light on the question of motivation at work from different angles. They include Maslow [4] and his theory of hierarchy of needs, Herzberg [5] and his two-factor motivation theory, McClelland [6] with his acquired-needs theory, Vroom [7] and his expectancy theory, Alderfer [8] and his ERG theory, Locke [9] and his goal setting theory, and finally, McGregor [10,11] with theory X and theory Y.

Herzberg, a pioneer in motivation theory, ascertained that work motivation is determined by two sets of factors: high-order need satisfaction (motivating factors) and low-order need satisfaction (hygienic factors) [12]. According to dual-factor theory people have two main sets of needs: a) hygienic needs, influenced by physical and physiological conditions at the workplace, which cannot motivate employees but can minimize dissatisfaction [13], and b) motivational needs, described by Herzberg as very similar to the higher-order needs of Maslow's hierarchy theory [14]. Hygienic needs are met with hygiene factors or inhibitors: supervision, interpersonal relations, working conditions, salary, company policy and administration, benefits and job security. They create satisfaction by fulfilling individuals' needs for meaning and personal growth [13]. When these factors, tied to the context of work environment, are unsuitable, job dissatisfaction ensues. Motivational needs are met by motivator factors or satisfiers: achievements, recognition, work itself, responsibility and personal growth. They are tied to the nature of the work itself. Factors leading toward job satisfaction are those that fulfil individual's need for self-actualization at work. The high intensity of hygiene factors does not necessarily leads to job satisfaction, but is instead related to a neutral state, characterized as neither satisfaction nor dissatisfaction. In contrast, the high presence of motivator factors normally leads to high job satisfaction [14].

Results of Herzberg’s studies reviled specific factors associated with job satisfaction and dissatisfaction. Since motivating factors (achievement, recognition, the work itself, responsibility, personal growth and advancement) did not overlap with hygienic factors (company policies, supervision, relationship with supervisor and peers, work conditions, salary, status, security), he draw two main conclusions. Satisfaction is not the opposite of dissatisfaction–instead, it only means no job satisfaction as such. The same is true for job dissatisfaction (absence of it does not immediately imply satisfaction). Only by eliminating dissatisfying job factors the performance at work will not be enhanced. In fact, even with best treatment of the hygienic factors, employees will be neither satisfied nor dissatisfied. It is only through boosting the motivating factors that a company can realistically expect enhanced motivation of their employees. Also, Herzberg’s results indicate that the effect to increased motivators lasts far longer that for the hygienic factors on employees’ attitudes [15].

Despite being the cornerstone of the modern motivation theory, Herzberg's approach fails to account for drastic changes beginning in the 1970. Namely, with the transition to knowledge economy and globalization, and with technology becoming the core engine of the development [16], new motivating factors surface, accompanied by different relationships among them. The purpose of this paper is to test whether Herzberg’s two—factor Hygiene and Motivation Theory is still as relevant today as in the 1960s. Since then, Herzberg’s two—factor Hygiene and Motivation Theory has been the subject of many critiques and further research [7,17–20]. In 1987 Herzberg again refuted any criticism and furthermore the key difference between the concepts of hygiene and motivation. He explained that the hygiene is a function of fear and punishment of failure in order to obtain external rewards. The concept of motivation is a function of personal growth and brings inner (personal) rewards such as an interesting and challenging work [2]. Herzberg emphasized that although at first it seems that the behavioural results coming from the changes in motivation vs. changes in hygiene are the same, this is far from true. This dynamics is far more complex and as such it brings extensive long-term consequences [2]. The hygiene function requires constant reinforcement and is reflected in the short-term results whereas the motivation is based on the need for personal growth and an inner generator, consequently the benefits are long-term. As a final reward motivate the personal growth, people are not required rewarded with increasingly higher prizes [2]. Herzberg in his commentary once again exposed the essence of its two factor system theory, namely considering that hygienics in the best case scenarios result in the absence of dissatisfaction in the workplace, while their absence creates dissatisfaction. Otherwise, people may be motivated by factors that are related with the content of the work, therefore motivators.

Several recent results sought to test if and to what extent the Herzberg’s theory still holds today, and which factors are more or less crucial [21–23]. An experiment on middle level managers in the context of the two-factor theory [22] came to the important result–namely, that both intrinsic and extrinsic motivational variables are important (when combined) in motivating middle level managers to achieve organizational goals. The study further concluded that it is important to consider a mixture of both when designing motivational strategies, given the importance of these employees in the survival of an organisation. Sithole and Solomon [24] applied Herzberg’s Two-factor Theory to teachers in secondary school in Botswana to test their job satisfaction. The aim of their research was to identify satisfying factors which would also improve their classroom performance vis-à-vis student achievements [24]. This work is significant as their findings suggest that teachers have quite a few dilemmas in terms to the two factor theory. According to hygiene factors, the teachers were mostly concerned with establishing good relations with school administrators, colleagues and students, adequacy of teaching resources and consumables, as well as their living conditions and salaries. In opposition to this, in terms of the motivating factors, results indicate that overall, teachers find teaching “satisfying” since they were not overly concerned with the pedagogical matters that form the core duties and responsibilities of teachers [24].

In this paper we present and analyse the results of a recent survey of Slovenian higher educated employees, relying on a sample of individuals carefully selected towards adequately reflecting the Slovenia's diversity in terms of age, job type (public or private) and place of residence. While money and prestige are still found to be relatively relevant factors across generations, our findings indicate that other contributing factors also play major substantial roles. In particular, we show that while not being strongly correlated with the demographic parameters, motivators are correlated among themselves in non-trivial ways.

The questionnaire used for the survey was based on Herzberg's original questionnaire from 1968. A novel step in our work was to analyse the correlations between motivators and inhibitors (or Herzberg’s hygienic factors), establishing their relative importance and the centrality of the components. We also examine to what extent are the demographic covariates important predictors among highly educated employees in Slovenia.

As opposed to above mentioned papers, our approach involves new data on a poorly surveyed country (Slovenia). Moreover, we analyse the correlations among factors via novel framework of network analysis, thus providing a new view point on this interesting problem. The choice of our methodology relies on searching for the most central motivators/inhibitors which are likely to be the “core” of motivating structure. Also, given Slovenia’s socialist past and capitalist present, our results shed new light on work motivation in a country of this type. As discussed above, most “traditional” motivation theories emphasize money (individual economic benefit) and prestige (honour, recognition in society) as the only true widely applicable motivators, at least in the Western societies [25,26]. However, equivalent results for post-socialist (ex Eastern block) countries are scarce, even though it is already clear that motivator structure in this case is different from that in the “old” capitalist countries.

## Methods

The sample of subjects was randomly selected from Slovenian Business Register, which contains the nationwide data on employers. A cover letter with instructions on how to complete the survey was sent to a selected sample of employees (or subjects or respondents). Subjects participated voluntarily, meaning that they were not financially compensated for the participation in the survey. Their participation was anonymous, not involving a name or any identifiable information about subjects.

Our main measuring instrument was a questionnaire based on the above discussed Herzberg's theory. In addition to the relevant socio-demographic data, the questionnaire consisted of questions regarding the factors that govern job satisfaction. For consistency, each factor’s examination was twofold: firstly in the form of a motivator and secondly in the form of an inhibitor (hygienic). The former measures the strength of the motivation (satisfaction) resulting from having/receiving more of something, while the latter reflects the magnitude of the inhibition (demotivation, dissatisfaction) resulting from having/receiving less of it. For instance, the topic on a person’s salary can be investigated as a motivator through question: “How important is higher salary to you?”. At the same time, the issue of salary can be examined as inhibitor by re-formulating the question as: “Would you mind having lower salary?”. The questionnaire consist of 30 + 30 questions (motivators + inhibitors)

When answering the question regarding the motivators, the subjects were instructed to think of a time when they were most motivated by reviewing an ideal past situation at work. Furthermore, they were asked to grade their answer using a scale, indicating how strong effect each motivator had on the respondent. The grading scale was defined from 0 (no effect on their work motivation) to 6 (maximal effect on their work motivation). On the other hand, when answering the questions regarding the inhibitors, the respondents were asked to think of a time when they were least motivated in the work environment and as previously, they graded their answers using a scale to indicate the influence of their job dissatisfaction. The scale was defined equivalently, meaning from 0 (no inhibition) to 6 (maximal inhibition).

The questionnaire was structured in a way to test the discrepancy between the same issues being posed as the motivators as opposed to being posed as the inhibitors, which represents the core of Herzberg’s theory. Observing the positive effect for a given factor posed as a motivator, does not necessarily result in the equally negative effect for the same factor when posed as an inhibitor. The English version of the questionnaire can be found in S1 File.

### Sample

The survey was sent to all employees in 50 public and private organizations in Slovenia, which hire higher educated employees. We received the survey correctly completed by 273 subjects. The analysis that follows focuses on these data.

32% of the respondents were employed in the public sector at the time the survey was conducted whereas the remaining 68% were employed in the private sector. 49.8% of them lived in a city (population exceeding 50,000), 27.5% in a town (population between 5,000 and 50,000), while the remaining 22.7% lived in the countryside (population below 5,000). 41.7% of the respondents earned 1,500€ net income per month or more, 34.1% between 1,000€ and 1,500€ per month, 18.3% earned between 700€ and 1,000€ per month, while the remaining 5.9% earned less than 700€ per month. In Slovenia, the net income is calculated by subtracting the income tax, public health insurance, social security and other minor deductions from the gross income. The age of the respondents varied from 26 to 75 years (mean 40.6 years ± 10.2 years).

**Fig 1:**
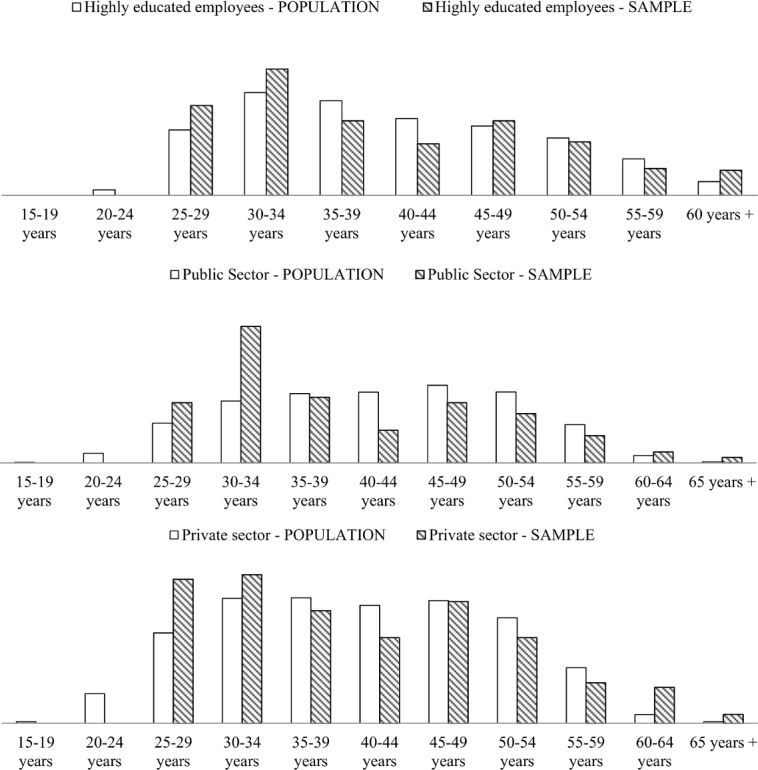
Comparison of the distributions for the entire Slovenian higher educated population and the same distributions for our sample. Panels, top to bottom: distribution of age; distribution of age in the public sector; distribution of age in the private sector.

To verify the representativeness of the sample, we make a comparison of socio-demographic parameters of the sample against the entire population. Fig 1 shows the distributions (histograms) of the employment considered over age. All histograms show that indeed the respondents were selected in a way which adequately reflects the general employment diversity of Slovenian higher educated population. On these grounds we believe that our results, described in the following section, are representative of the entire Slovenian higher educated population.

### Ethics statement

All subjects consented to the participation in the survey. Their personal anonymity was preserved (although the relevant data are known to the authors). The study involves no ethical concerns, and it was approved by the Academic Board of the School of Advanced Social Studies in Nova Gorica, Slovenia.

## Results

The subjects described above were surveyed using the questionnaire described in the Introduction and available as S1 File. For each of the 273 subjects, 60 grades from 0 to 6 were obtained, corresponding to 60 questions (30 motivator versions and 30 inhibitor versions). Below we present the analysis of the data. Complete data are provided in S2 File.

### Bivariate analysis

There is a significant association between sectors of employment and salary category *χ* ^2^(2) = 9,842, *p* < 0,05. A higher percentage of the employees from the public sector (70%) earn less than 1,500€ per month compared to the private sector (50%). This picture changes completely when considering earnings exceeding 1,500€: 29% from the public sector and 50% from the private sector. There is also a significant association between the age category of employees and salary category; *χ* ^2^(2) = 80,303, *p* < 0,001. There are higher percentage of employees under 40 years of age that earn less than 1,500€ per month (81%), compared to those over 40 years of age (30%). Considering the earnings over 1,500€ per month, only 19% of subjects were under, while 70% are over 40 years of age.

**Fig 2:**
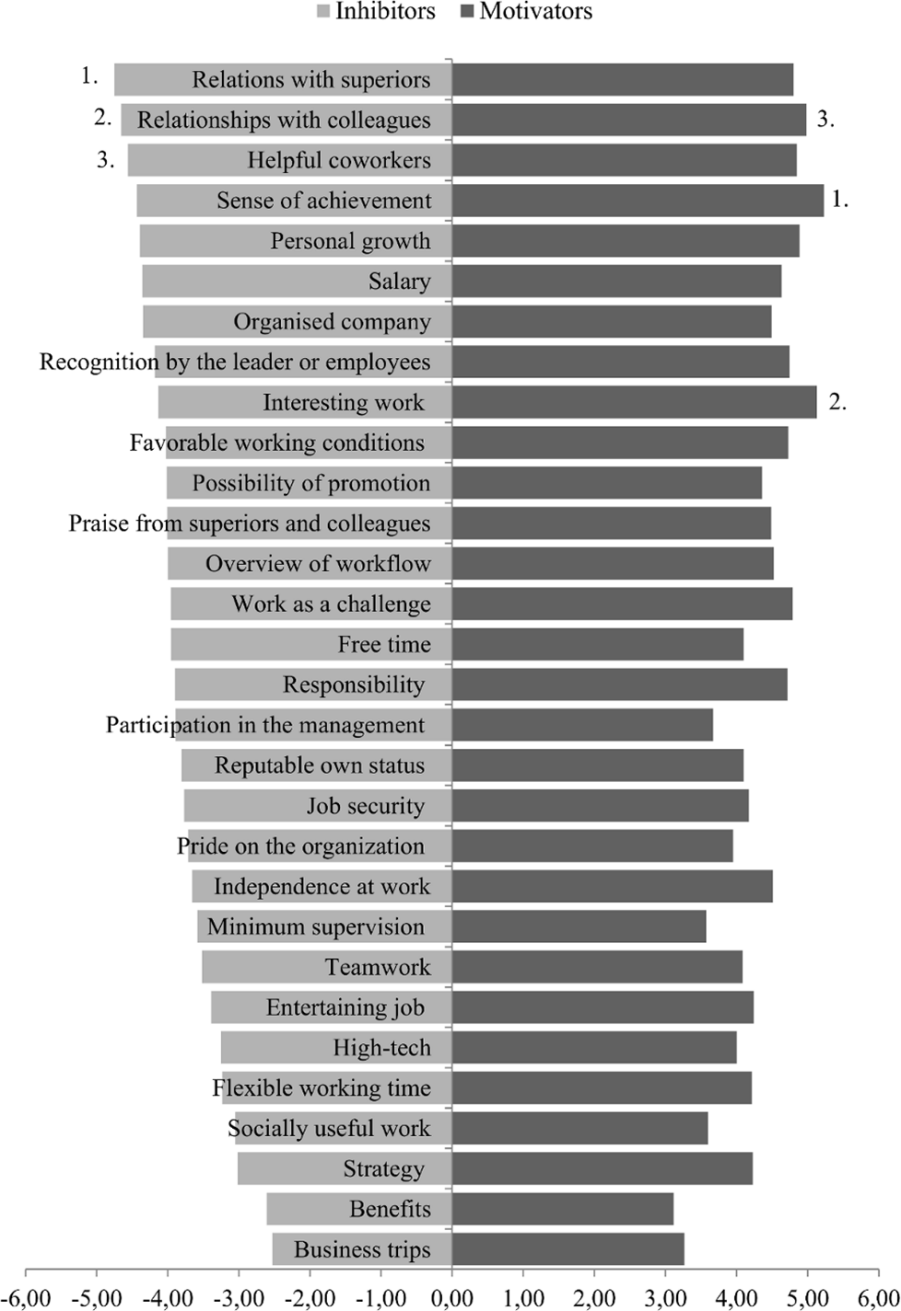
Evaluation of all 30 factors with names indicated. Lighter (darker) grey bars pointing towards left (right), i.e. in the negative (positive) direction, illustrate the average grade of a factor when understood as an inhibitor (a motivator). Factors are organized by descending order defined by the decreasing average inhibitor grade. First three motivators are indicated.

Next, we examine the evaluation of factors (motivators and inhibitors), regardless of the socio-demographic parameters, by considering the average grades that each of them received. Results are shown in Fig 2 –for each factor, the size of the lighter bar pointing towards left reports about the average grade as inhibitor, while the size of the darker bar pointing towards right indicates the average grade as motivator. The factors are organized top to bottom by decreasing average grades as inhibitor.

The top three motivating factors are sense of achievement (high quality work execution), interesting work and good relations with the colleagues. On the other hand, top three inhibitors are bad relations with the superiors, bad relations with the colleagues and unhelpful colleagues. Only “relations with the colleagues” is evaluated within first three for both motivators and inhibitors. In fact, it is clear from the figure that the order of motivators only approximately coincides with the order of inhibitors. This indicates that that strong motivation due to presence of something, is not necessarily correlated with strong inhibition coming from absence thereof. We also note that the business trips and various employment benefits are the two lowest evaluated factors, both as motivators and inhibitors. We conclude from these information that personal and in fact interpersonal relations at work seem to matter much more than usual money and prestige factors such as salary or business trips.

### Correlation analysis

**Fig 3:**
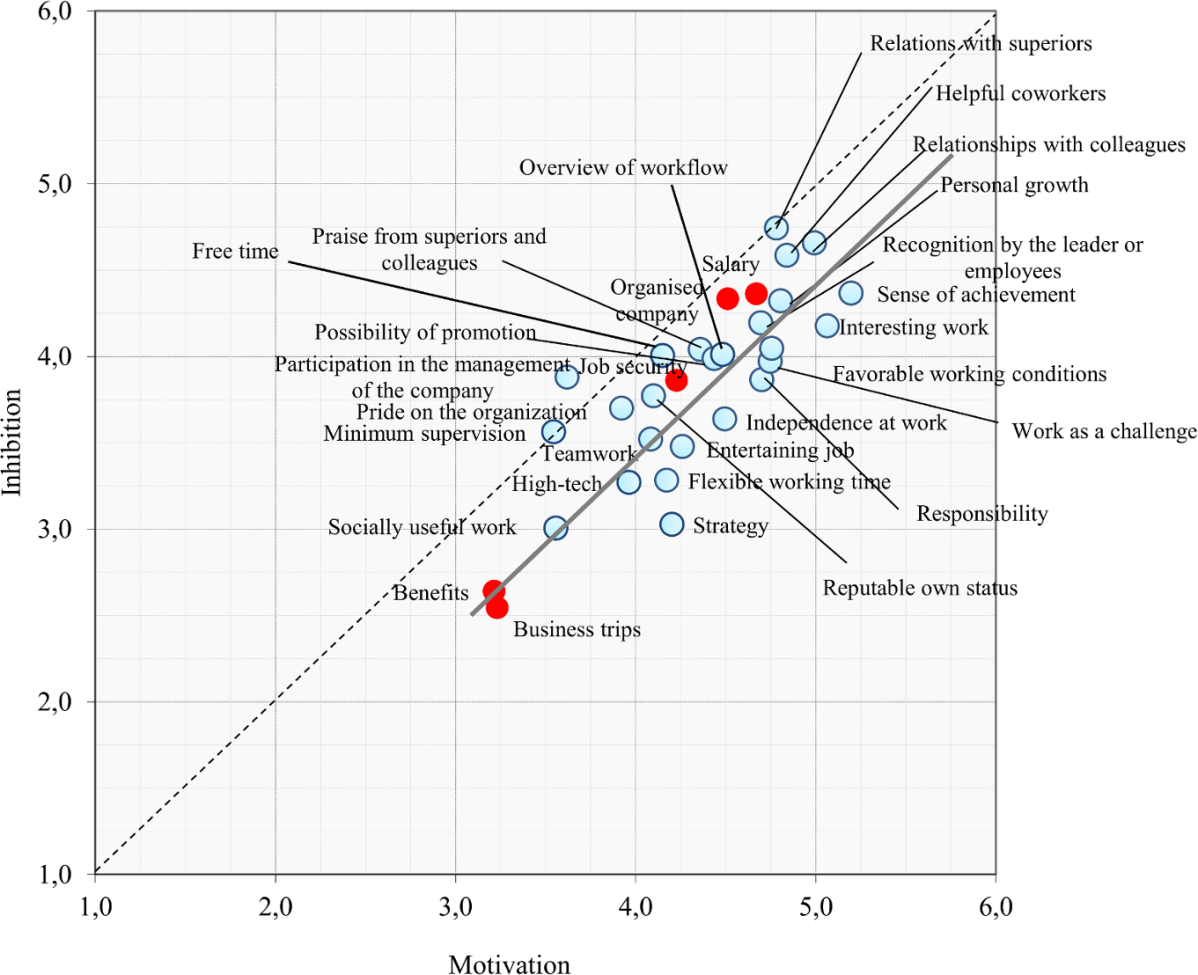
Scatter plot of factors—motivation vs. inhibition. Each point represents one of the 30 factors, in way that the point's x (y) coordinate corresponds to the motivation (inhibition) intensity for that factor. Five factors capturing money and prestige are indicated. Diagonal line is indicated, along with the scatter plot linear fit line.

So how much precisely is the average motivation grade (intensity) related to the average inhibition grade? We begin by constructing a scatter plot shown in Fig 3, where each factor is described by two coordinates: the intensity of motivation on x axis, and the intensity of inhibition on y axis.

Motivation and inhibition are rather strongly correlated, almost in a linear relationship, which in general is expected: more the presence of something motivates us, more we will miss it when absent. Nevertheless, the correlation is still far from perfectly linear, indicating that certain factors matter more as motivators, while others matter more as inhibitors. As already observed, top rated motivators/inhibitors mostly concern personal and interpersonal dimensions, such as relationships (among colleagues and with superiors). Also highly evaluated are recognition (from colleagues and superiors), sense of achievement, personal growth, interesting and challenging job, organized company with structured promotional scheme and favourable working conditions. On the other hand, least important motivators and inhibitors are business trips, benefits, socially useful work, high-tech equipment, teamwork, supervision, strategy, vision and mission of the organization, entertaining work, and pride on the organization. We identify in show in red five factors that best capture both money and prestige: employment benefits, business trips, job security, high salary and well organized company. It seems that they overall do not play a major role, in fact, benefits and business trips are the two lowest evaluated factors.

We now investigate the linear fit line shown in the figure. In principle, one would expect it to overlap with the diagonal, which would imply that motivation intensities are equally strong as the corresponding inhibition intensities. Interestingly, this linear fit line actually lies parallel and below the diagonal. This amounts to a consistent shift (about 0.4 in our scale) towards stronger motivation and weaker inhibition. That is to say that on average, motivation intensity for a given factor is slightly stronger than the corresponding inhibition intensity for the same factor. In other words, the respondents are in general somewhat happier with having something, than disappointed by not having it. We interpret this as a manifestation of a global optimism (positive motivation) present in the Slovenian society. This leads the employees to look at the “bright side of things”, and see more clearly the benefits of having something than the drawbacks coming from not having it.

Next we are interested to check if there is any difference between place of residence, sectors of employment and salary category in relation to perception of important motivators and inhibitors.

Top three motivating factors for both rural and non-rural inhabitants are the sense of achievement, interesting work and relationships with colleagues (see Table 1). Relations with colleagues are important both as a motivator and inhibitor. If we focus on inhibitors on the other hand, we see that top three inhibitors are bad relations with superiors, bad relationships with colleagues and bad (unhelpful) colleagues, which are same for all three categories of respondents.

**Table 1:** Top three motivators and inhibitors in relation with the place of residence.

City		Town		Countryside	
Motivators	Inhibitors	Motivators	Inhibitors	Motivators	Inhibitors
Sense of achievement at the quality of completed work.	Bad relations with the superiors.	Sense of achievement at the quality of completed work.	Bad relations with the superiors.	Sense of achievement at the quality of completed work.	Bad relations with the superiors.
Interesting work that motivates and completes me.	Bad relations with the colleagues.	Interesting work that motivates and completes me.	Bad relations with the colleagues.	Good relations with the colleagues.	Bad relations with the colleagues.
Good relations with the colleagues.	Unhelpful colleagues.	Personal growth.	Unhelpful colleagues.	Interesting work that motivates and completes me.	Unhelpful colleagues.

Most important motivating factors that both public and private sector employees are sense of achievement, interesting work and relationships with colleagues (see Table 2). Public sector employees are then motivated also by helpful colleagues and good relations with colleagues.

**Table 2:** Top three motivators and inhibitors depending on the employment sector.

Public sector		Private sector	
Motivators	Inhibitors	Motivators	Inhibitors
Sense of achievement at the quality of completed work.	Bad relations with the superiors.	Sense of achievement at the quality of completed work.	Bad relations with the superiors.
Interesting work that motivates and completes me.	Bad relations with colleagues.	Interesting work that motivates and completes me.	Bad relations with the colleagues.
Good relations with the colleagues.	Unhelpful colleagues.	Good relations with the colleagues	Unhelpful colleagues.

Top three motivating factors for all salary categories are sense of achievement, good relationships with colleagues and interesting work (Table 3). Top three inhibitors on the other hand are all connected to the human aspect of the working environment, such as bad relations with superiors, bad relations with colleagues and unhelpful colleagues.

**Table 3:** Top three motivators and inhibitors depending on the salary category.

Less than 1,000€		From 1,000€ to 1,500€		1,500€ or more	
Motivators	Inhibitors	Motivators	Inhibitors	Motivators	Inhibitors
Sense of achievement at the quality of completed work.	Bad relations with the superiors.	Interesting work that motivates and completes me.	Bad relations with the superiors.	Interesting work that motivates and completes me.	Bad relations with the superiors.
Good relations with the colleagues.	Bad relations with the colleagues.	Sense of achievement at the quality of completed work.	Bad relations with the colleagues.	Sense of achievement at the quality of completed work.	Bad relations with the colleagues.
Interesting work that motivates and completes me.	Unhelpful colleagues.	Good relations with the colleagues.	Unhelpful colleagues.	Good relations with the colleagues.	Unhelpful colleagues.

From tables above it appears there is not much variability regarding top three motivators and inhibitors depending on the considered parameters (place of residence, employment sector and salary). In sum the human touch play a predominant role as inhibitor in the work environment, while the intrinsic nature of the work is the top motivator.

### Network analysis

We conclude this section by extending the above correlation analysis to study of the correlation network among various factors. To our best knowledge, this is a first such study in the context of motivation theory.

We begin by defining the correlation of two factors (considered as either motivators or inhibitors), as the value of the usual Spearman correlation between them, obtained by computing over all respondents grades. High correlation indicates that high grades for factor A typically come with equally high grades for factor B, whereas low correlation means the opposite. A set of factors that is mutually well correlated allows for employees to get easily motivated (or inhibited) for all of them, starting with getting motivated (or inhibited) for just one of them.

Now, a strong correlation between a motivator pair A-B and between B-C does not necessarily imply any correlation between motivators A and C. That means that motivators are not all correlated with all, but instead, one can identify and study certain patterns of correlations among motivators. That is to say that these correlation patterns in general compose a complex network, whose structure can be studied via standard network analysis. Actually, network analysis in the recent decades established itself as a powerful framework for examining complex systems in nature and society, and has contributed significant advancements in fields as diverse as physics, biology, sociology and psychology [27–29]. The most propulsive sub-fields such as social network analysis and bioinformatics are benefiting from large amounts of data that is increasingly becoming freely available. The social network perspective focuses on relationships among social entities and is an important addition to standard social and behavioural research, which is primarily concerned with attributes of the social units [27–29].

**Fig 4:**
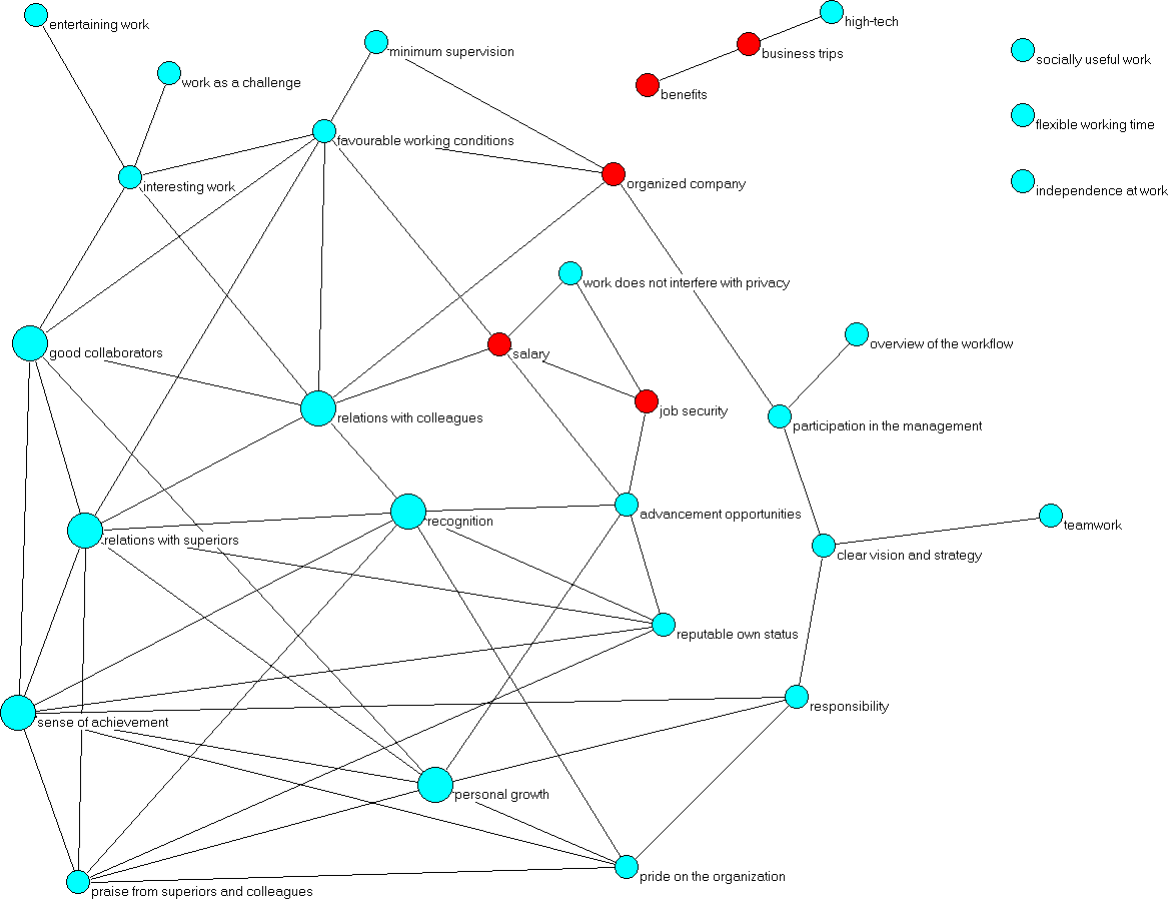
Correlation network among the motivators. Nodes (cyan and red) represent motivators (denoted by names), and a link between a pair of motivators indicate, that the correlation between that motivator pair is at least 0.4.

**Fig 5:**
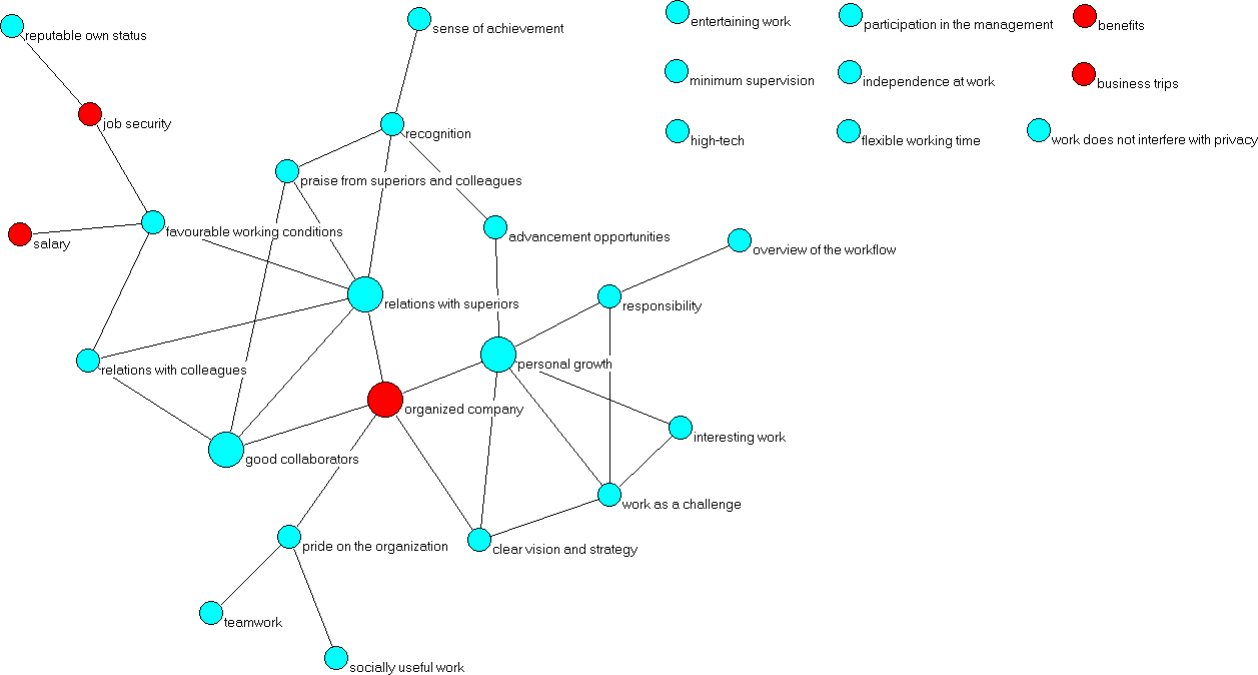
Correlation network among the inhibitors, done equivalently to Fig 4.

In this paper we take a slightly different approach, and construct our network as follows: 30 motivators are modelled as 30 nodes of a network (graph). A link between a pair of nodes (motivators) exists if the Spearman’s correlation between them is stronger than 0.4. The graphical representation of this network is shown in Fig 4. The same is done of inhibitors, and the resulting network is shown in Fig 5. The threshold correlation value of 0.4 is selected to include only the most relevant motivator/inhibitor pairs, leaving out weakly correlated ones. These correlation networks capture structure of correlation patterns among motivators and inhibitors.

We conduct a simple network analysis by emphasizing in Figs 4 and 5 the factors that are most central in each network (shown via arrows). For the centrality measure we take a combination of degree (number of neighbours) and closeness centrality (the accessibility of the network seen from a given node). That is to say that each node identified as having high centrality is both well connected to its local neighbourhood (high degree), and also on average relatively close (in terms of network distance) to all other network nodes (high closeness centrality).

The most central motivators are again related to interpersonal dynamics in addition to the desire to work in a well-organized company. Similarly, the most central inhibitors are also mostly related to the interpersonal relationships at work. In both figures we denoted in red the factors related to money and honour–yet again, they mostly seem not to play a central role.

To explain the added value of the network approach to our problem, we recall that a link between two factors means that they are strongly correlated. An employee with strong feelings about a certain motivator/inhibitor is likely to soon have similarly strong feelings motivators/inhibitors linked to it, and perhaps ultimately about those linked to them. Now, if this initial motivator/inhibitor is a very central node, it is easier for motivation/inhibition to spread to other nodes, potentially the entire network. In other words, a good strategy for an employer who wants to motivate an employee is to satisfy his/her wishes regarding one of the central motivators, and the motivation for other factors is likely to emerge spontaneously.

Interestingly, we also note that the inhibitor correlation network is globally better connected than the motivator correlation network (more links in the network). That is to say, inhibitors are more entangled among them, in contrast to motivators, which are more isolated among them. This can be related to the fact that it is still easier for people to get generally disappointed with work than to get generally enthusiastic about it: failing at one of the central inhibitors is likely to spark an avalanche of disappointments, easily leading to global demotivation. In contrast, getting motivated about one factor, even if it is a central one, is still no guarantee that the person will soon be overall satisfied at work.

## Discussion

We presented a systematic analysis of the interplay among the factors that motivate and inhibit people for work, done for a representative sample of employees in an ex-socialist country. Using a novel approach of complex networks, we showed for the first time the intricate web of factors contributing to the satisfaction or dissatisfaction of the employees. Far from Slovenians being driven solely by financial or reputation benefits, our results show that it is hard to pinpoint a single key motivator as well as a single key inhibitor. Instead, our subjects seem to be driven by various combinations of factors, that mostly, but by no means exclusively, have to do with personal and interpersonal dimensions of the workplace life. Grounds for the optimal working conditions thus seem to consist of good relations with the colleagues and the superiors, opened opportunities for personal growth, and good organization within the private company or the public entity. In addition, through scatter plot analysis we revealed, somewhat to our surprise, a quantitative evidence for a general optimism about professional life in Slovenia. In fact, the value of the shift of the linear fit line (around 0.4) might be a value characteristic for Slovenia, which opens the question about this value for other countries. More interesting would be to examine the correlation between these “shift values” and the other parameters that can be computed for a country’s economy, for example gross domestic product of buying power.

Coming back to the Herzberg's theory discussed in the Introduction, we conclude that its basic framework and the bulk body of his theory still stand. Nevertheless, we believe our paper to provide a significant novel contribution.

We should also acknowledge the limitations of our study. Sampling method is the first among them, since our sample is to some extent a convenience sample, and as such is a type of non-probability sampling technique. Secondly, we took into consideration only highly educated employees from both private and public sector, due to low accessibility of lesser educated employees. This however leaves the question of their motivation and inhibition still open. Thirdly, we were dealing only with the circumstances in Slovenia, leaving open the issue of other countries, both ex-socialist and “old” capitalist. Fourthly, the absence of longitudinal data (measured over a certain amount of time) would enable to eliminate the factor of age stratification, which is due to the personal development of each individual. It would also allow the study of changes of the factor intensities over time and over person’s life.

Using network analysis we wanted to show that not only factors that are visible at first sight (evaluated) are the sole important conditions for employees to stay motivated at work. We believe that of the same importance if not higher is the structure of the hierarchy of the importance of motivators and inhibitors. It is also to be emphasized that the engagement of a specific factor in orchestra as a hole may play a much more crucial role orchestrating the other factors even though is not important itself, which can be revealed exactly via approaches suitable for complex systems, such as network analysis. It is our hope that studying human societies and the theories describing them as complex systems could trace new avenues towards better understanding and managing our civilization.

## Supporting Information

S1 File The English version of the questionnaire that was used in the survey. (PDF)

S2 File The Excel table with the complete survey data. (XLSX)
