# Supplementary material for: What Motivates Us for Work? Intricate Web of Factors beyond Money and Prestige
Source: PLoS One. 2015 Jul 15;10(7):e0132641. doi: 10.1371/journal.pone.0132641 (PMC4503444; doi:10.1371/journal.pone.0132641)
Supplement: S1 File — (PDF) [file pone.0132641.s001.pdf]

What motivates us for work? Intricate web of factors beyond money and prestige

Nadja Damij (1), Zoran Levnajić (1,2,3), Vesna Rejec Skrt (4), Jana Suklan (2,5)

(1) Faculty of Information Studies in Novo mesto, Novo mesto, Slovenia
(2) School of Advanced Social Studies, Nova Gorica, Slovenia
(3) Faculty of Computer and Information Science, University of Ljubljana, Slovenia
(4) Varsi d.o.o., Stegne 35, Ljubljana, Slovenia
(5) Interdisciplinary Doctoral Study Programme in Statistics, University of Ljubljana, Slovenia

Supplementary information - Questionnaire

Q1. In the first part of the questionnaire 30 factors are listed.

Think about a time in your life when you were mostly motivated at work. For each factor listed in the table below, please indicate if and how much it affected your motivation height.

Focus on an ideal situation, not the actual situation in the company in which you are currently working.

Factors rated from 1 (factor does not affect my work motivation at all) to 6 (factor COMPLETELY affect my work motivation).

	1	2	3	4	5	6
Sense of achievement						
Recognition by the leader or employees						
Overview of the workflow						
Responsibility						
Advancement opportunities						
Personal growth						
Clear vision and strategy of the company						

Organized company						
Minimum supervision						
Good relations with superiors						
Favourable working conditions						
Good salary and awards						
Good relations with colleagues						
Work does not interfere with my privacy						
Reputable own status						
Employment security						
Independence at work						
Business trips						
Socially useful work						
Pride related to the organization						
Interesting work						
Teamwork						
Participation in the management of the company						
Praise from superiors and colleagues						

Work as a challenge						
Entertaining work						
Flexible working time						
Good collaborators						
High-tech technology						
Various benefits						

Q2. In the second part of the questionnaire there are re-listed 30 factors. It is very important that you do not think about how you evaluated the first set, it's not the same thing. Nor is it necessary that the same factor also affects your frustration and your motivation. For example, factor X (eg. a challenge, working conditions ...) it is possible that cause you high dissatisfaction if not present; while if present it does not motivate you because you take them for granted.

Think about a time in your life when you were mostly dissatisfied at work. For each factor listed in the table below, please indicate if and how much it affected your level of dissatisfaction.

Factors rated from 1 (factor does not affect my work dissatisfaction at all) to 6 (factor COMPLETELY affect my work dissatisfaction).

	1	2	3	4	5	6
No sense of achievement						
No recognition by the leader or employees						
No overview of the workflow						
No responsibility						
No advancement opportunities						
No opportunities for personal growth						

No clear vision and strategy of the company						
Not a well-organized company						
Strict supervision						
Bad relations with superiors						
Unfavourable working conditions						
Poor salary and no awards						
Bad relations with colleagues						
Work interfering with my privacy						
Non-reputable own status						
No employment security						
No independence at work						
No business trips						
Socially not useful work						
No pride related of the organization						
Not an interesting work						
No teamwork						
No option about participation in the management						

No praise from superiors and colleagues						
Don’t see work as a challenge						
Boring work						
Non flexible working hours						
Bad collaborators						
No high-tech technology						
No benefits						

For the end I will ask you some demographic information. The data collected will be analysed and a summary appear only in summary form and used exclusively for scientific research purposes.

Q3 - Year of Birth

Q4 - Living in:

Major cities	Small towns	Village or rural areas

Q5 - The level of the last completed education:

High school	University or more

Q6 - The average net monthly earnings:

up to € 700.00	up to € 1,000.00	up to € 1,500.00	1,500 or more €

Q7 - Employed in:

public sector	private sector
